# A Randomized Trial Comparing Short versus Prolonged Hemostasis with Rescue Recanalization by Ipsilateral Ulnar Artery Compression: Impact on Radial Artery Occlusion—The RESCUE-RAO Trial

**DOI:** 10.1155/2020/7928961

**Published:** 2020-10-23

**Authors:** Dmitrii V. Ognerubov, Alexander Sedaghat, Sergey I. Provatorov, Andrey S. Tereshchenko, Olivier F. Bertrand, Ivo Bernat, Goar K. Arutyunyan, Olga A. Pogorelova, Maria I. Tripoten, Tatyana V Balakhonova., Anatoliy N. Samko, Evgeny V. Merkulov

**Affiliations:** ^1^National Medical Research Center of Cardiology, Ministry of Health of Russia, Moscow, Russia; ^2^Medizinische Klinik und Poliklinik II, Universitätsklinikum Bonn, Bonn, Germany; ^3^Quebec Heart-Lung Institute, Quebec, Canada; ^4^University Hospital and Faculty of Medicine Pilsen, Pilsen, Czech Republic

## Abstract

**Background:**

Despite the enormous benefits of radial access, this route is associated with a risk of radial artery occlusion (RAO).

**Objective:**

We compared the incidence of RAO in patients undergoing transradial coronary angiography and intervention after short versus prolonged hemostasis protocol. Also we assessed the efficacy of rescue 1-hour ipsilateral ulnar artery compression if RAO was observed after hemostasis. *Material and Methods*. Patients referred for elective transradial coronary procedures were eligible. After 6 F radial sheath removal, patients were randomized to short (3 hours) (*n* = 495) or prolonged (8 hours) (*n* = 503) hemostasis and a simple bandage was placed over the puncture site. After hemostasis was completed, oximetry plethysmography was used to assess the patency of the radial artery.

**Results:**

One thousand patients were randomized. Baseline characteristics were similar between both groups with average age 61.4 ± 9.4 years (71% male) and PCI performed on half of the patients. The RAO rate immediately after hemostasis was 3.2% in the short hemostasis group and 10.1% in the prolonged group (*p* < 0.001). Rescue recanalization was successful only in the short group in 56.2% (11/19); at hospital discharge, RAO rates were 1.4% in the short group and 10.1% in the prolonged group (*p* < 0.001).

**Conclusion:**

Shorter hemostasis was associated with significantly less RAO compared to prolonged hemostasis. Rescue radial artery recanalization was effective in > 50%, but only in the short hemostasis group.

## 1. Introduction

Radial access (RA) has become the gold standard for coronary angiography (CAG) and percutaneous coronary interventions (PCI) within the last decade. The use of this access is associated with reduced rates of access site complications, MACE, and improved clinical outcomes, including survival [1–3]. In addition to clinical advantages, RA is linked to socioeconomic benefits including reduced in-hospital stays and costs [2]. The main convenience of RA results from the superficial location of the radial artery, its smaller diameter, and the fact that the radial artery can be easily compressed while allowing patient mobility [4]. A relevant downside of RA is the relatively high incidence of radial artery occlusion (RAO). This mostly asymptomatic complication can be found in 1%–30% of patients after radial access [5–9]. Although clinical symptoms are uncommon and critical ischemia is rarely encountered [10], the presence of RAO makes this artery unsuitable for repeated access or other clinical uses including arterial grafting in coronary bypass surgery or in the context of arteriovenous fistula surgery for dialysis. Prevention, early detection, and effective treatment of RAO are therefore of clinical importance in maintaining patency of this access route. Adequate compression should be applied to reduce local bleeding complications and also to achieve adequate hemostasis. The compressive pressure should be reduced earlier, but with careful monitoring for local bleeding complications or rebleeding [11, 12]. In this study, we aimed to compare the incidence of RAO in patients undergoing both diagnostic CAG and PCI using 2 different regimens of radial artery hemostasis. We further compared the efficacy of ipsilateral ulnar artery compression for the treatment of early RAO in both groups.

## 2. Material and Methods

### 2.1. Study Cohort and Study Design

**Figure 1:**
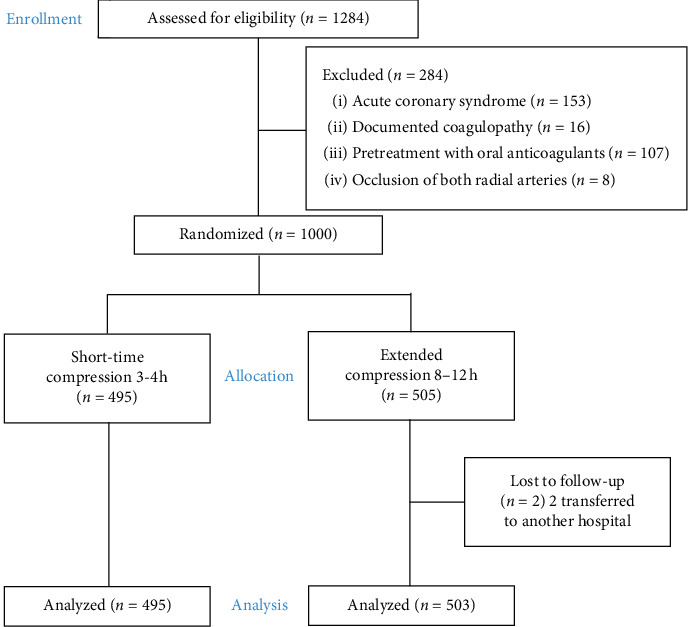
Study flow diagram.

The RESCUE-RAO Trial (a randomized trial comparing short versus prolonged hemostasis with rescue recanalization by ipsilateral ulnar artery compression-impact on radial artery occlusion) was performed at a large cardiology center in the Russian Federation. Patients referred for diagnostic CAG or PCI using RA were enrolled. The study flow diagram is seen in Figure 1.

Patients had to be older than 18 years old and needed to provide written informed consent to participate in this study. Due to more aggressive preclinical treatment and less predictable anticoagulation, patients with acute coronary syndrome as well as patients with documented coagulopathy or under treatment with oral anticoagulants were excluded. The study protocol and design were approved by the local institutional review committee and ethics committee of the National Medical Research Center of Cardiology in Moscow. All patients signed informed consent to participate in the study before the inclusion. Patients were pretreated prior to catheterization with acetylsalicylic acid (125 mg daily) and clopidogrel (75 mg daily). Anatomy of both radial arteries was evaluated by ultrasound before the procedures in all patients. In the short group, pressure dressings were applied for a mean of 3 hours, according to the previous unpublished pilot study, whereas in the prolonged group, pressure dressings were applied for 8 hours according to the local clinical experience [13].

The primary endpoint of this study was defined as the presence of 24-hour RAO after the procedure confirmed by oximetry-plethysmography test and duplex ultrasound. Secondary endpoints included (a) rate of successful recanalization of initial RAO and (b) rate of local bleeding and hematoma.

RAO was defined as negative oximetry/plethysmography test [14] (absence of plethysmographic signals from pulse oximeter placed on the index during ulnar compression) immediately after the end of hemostasis, that is, when the pressure dressing was taken off. Duplex sonography was performed in patients with suspected RAO by the oximetry/plethysmography test. All patients who had suspected RAO were examined with duplex sonography 24 hours after the procedure. Local upper extremity hematoma was categorized according to the EASY scale [15]. Bleeding from the access site was defined as the bleeding that occurred 3 hours after the intervention and which required a reexamination by a physician and the replacement of the dressing.

### 2.2. Procedural Specifics

Preprocedural duplex ultrasound was performed in all patients. Vessel patency, tortuosity, and the internal diameters were examined with a standard ultrasound device (Phillips IU-22, Philips N.V., Netherlands) using a linear probe L3-9. The images were obtained by dedicated vascular ultrasonographists, who were blinded to the study.

Radial artery catheterization was performed according to the Seldinger technique as previously described [12, 16]. Local anesthesia was performed by a subcutaneous injection of 2% lidocaine at the puncture site. After insertion of a transradial access sheath (Radifocus Introducer II, Terumo, Europe N.V.), 5,000 IU of unfractionated heparin (UFH) was injected for diagnostic coronary angiography, whereas 100 IU/kg UFH was used for PCI patients. Procedures were performed via 6Fr catheters. A spasmolytic cocktail was not routinely given in this study. Additional nitroglycerin was given by the discretion of the treating physician. In this study, radial artery hemostasis was achieved through compression of the radial artery using a pressure dressing with a roller placed directly in the puncture site [7, 8].

### 2.3. Detection and Treatment of the Radial Artery Occlusion (RAO)

**Figure 2:**
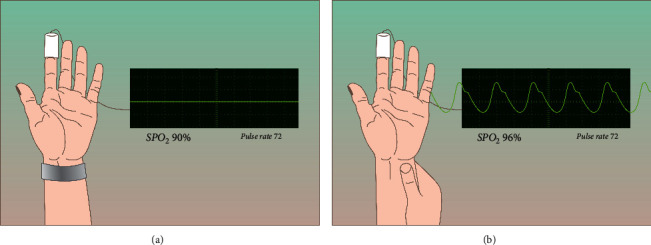
Scheme of radial artery recanalization. (a) Compression of the ulnar artery with TR band for one hour (no plethysmographic waveform (RAO)). (b) Repeating pulse oximeter test and recording the appearance of plethysmographic waveform indicating that recanalization of RAO was successful.

**Figure 3:**
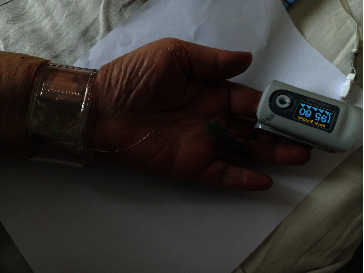
Compression of the ulnar artery with TR band with radial flow restoration (the appearance of plethysmographic waveform).

After removal of the compression bandage, patients were evaluated for RAO with oximetry/plethysmographic test [16, 17]. The pulse oximetry curve at the index finger was recorded with a standard pulse oximeter (Armed YX302, Russia). A positive result was defined as the detection of oscillations on the oximetry curve during compression of the ipsilateral ulnar artery [18, 19]. A negative result, that is, suspected RAO, was defined as the absence of the oximetry curve [19]. After initial testing, a second oximetry/plethysmography test was repeated 24 hours after the procedure in all patients. In patients with RAO, additional ultrasound of the forearm arteries was performed. When RAO was detected after initial hemostasis, we performed 1-hour compression of the ipsilateral ulnar artery. At the point of best pulsation 2-3 cm above the wrist, a TR band (Terumo, Japan) was inflated with 15–18 cm^3^ of air. Complete occlusion of the ulnar artery was confirmed by the absence of the curve on a pulse oximeter located on the index finger. Excess air was removed so as not to hinder venous outflow and not to create additional pressure on the occluded radial artery and surrounding wrist tissues. One hour later, the ulnar artery was released and the oximetry/plethysmography test was repeated. Successful recanalization was noted upon return of the signal on the oximetry/plethysmography test. The scheme of the recanalization technique is presented in Figures 2 and 3.

### 2.4. Statistical Analysis

For statistical processing of the results, we used the statistical program *R* 3.4.4 software (R Core Team [2014], Vienna, Austria). For all patient quality characteristics, the relative frequency of all possible parameter values was indicated. When comparing different groups, the following statistical criteria were used: (1) Student's *t*-test for independent samples was used for quantitative characteristics distributed according to normal law; (2) for quantitative characteristics that have an abnormal distribution, the Mann-Whitney test was used; (3) differences among categorical variables were evaluated using the chi-square test or Fisher exact test. Differences were considered statistically significant at *p* < 0.05.

The sample size calculation was performed using a superiority design with the assumed incidence of the primary endpoint of RAO of 8,8% in prolonged hemostasis [8] and 2.8% (reference data derived from local registry) in short-time hemostasis. Overall, 494 patients per group (total = 988) were deemed adequate to achieve 90% power considering an alpha error of 0.01, assuming a 5% rate of crossover from short-time hemostasis to prolonged hemostasis due to bleeding or hematoma [20].

## 3. Results

From 2015 to 2018, from 1284 all-comers patients screened, a total of 1000 patients undergoing diagnostic CAG or PCI were randomized with sealed envelopes in two groups. Patients in both groups were randomized before sheath removal to receive either short (*n* = 495) or prolonged hemostasis (*n* = 505). Two patients from the extended group were transferred to another hospital and could not be evaluated.

As for clinical and procedural characteristics, no significant differences were observed between the two groups (Tables 1 and 2). Overall, patients were 61.4 ± 9.4 years of age and the majority were male (70.9%). Among concomitant diseases, arterial hypertension was found in 90.4% of cases, whereas dyslipidemia and diabetes mellitus were present in 28.3% and 22.5%, respectively. Smokers accounted for 25.3% of all patients.

The RAO rate immediately after hemostasis was 3.2% in the short hemostasis group and 10.1% in the prolonged group (*p* < 0.001). Rescue recanalization was successful only in the short group 11/19 (56.2%) versus none in the prolonged group (*p* < 0.001). The primary endpoint of 24-hour RAO occurred in 5.8 % (58/998) of patients. The incidence of RAO was significantly lower in patients undergoing short-time hemostasis (1.4% versus 10.2%, *p* < 0.001) (Table 3).

The frequency of bleeding from the access site in the groups with short and prolonged hemostasis was similar (0.8% and 0.8%, resp., *p*=1.0). In addition, no differences in the incidence of minor hematoma (EASY grade 1–2) were seen (2.2 % versus 2.2%, *p*=0.79). More pronounced access site hematoma (EASY grade 3) occurred only in 2 cases with prolonged hemostasis (0.4 %) and in one case of the short group (0.2%). None of the patients developed major access site hematoma (EASY grade 4-5) and needed surgical therapy or transfusion.

## 4. Discussion

The results of our randomized study demonstrate a statistically, significant, and clinically relevant reduction in the incidence of RAO detected 24 h after the procedure using short-time compression after radial access for both CAG and PCI. Rescue recanalization via ipsilateral compression of the ulnar artery was effective only in the short-time compression group. At the same time, there were no differences regarding bleeding or the development of hematoma at the access site between the 2 regimes.

With the ascent of radial artery access, prevention, and management of radial artery, occlusion has become a relevant issue [7, 12, 17]. Overall, our results regarding RAO are compared well with other studies published on this topic. The incidence of RAO varies greatly from 1.1 to 33% [7, 9, 17, 21]. Whereas Uhlemann et al. reported a relatively high incidence of RAO with 33.1% using 6 F introducers [9], RAO occurred in 5.8% of our patients. In contrast to our analysis, the authors used a commercially available dosage compression device. In the presented analysis, hemostasis devices, such as the Terumo TR Band, were not routinely used. Although the application of the air compression device has been found to be associated with a lower incidence of RAO [16], the additional cost of these devices is a relevant limitation regarding their use, especially in developing countries. In addition, the protocol, which has to be applied, has several limitations, including the need for larger involvement of the postprocedural care team and repeated evaluation of radial flow. Given the use of a simple pressure dressing, our results indicate the safety and feasibility of this approach with acceptable rates of RAO as well as bleeding [8].

Additionally, recanalization via ipsilateral compression of the ulnar artery led to a successful recanalization in 56.2%, only in those patients undergoing short-term compression. Otherwise, recanalization could not be achieved in any patient undergoing traditional hemostasis, while in the group of short hemostasis, 56.2% with RAO in short hemostasis were recanalized (*p* < 0.001). It remains possible that even more prolonged use (> 1 h) of ipsilateral ulnar artery compression could have a better result on the RAO recanalization rate. Bernat et al. showed comparable success of recanalization in 71% (5 out of 7) in a group of 5000 IU of heparin and 31% (4 out of 13) in 2000 IU heparin group, but they evaluated only patients after CAG [22]. In contrast, Tian et al. recanalized 98.9% of RAO, which is likely to be linked to prolonged manual compression of the ulnar artery in their study, which was performed for 4.1 ± 1.2 h [23]. In clinical routine, there are several potential reasons for the failure of recanalization of the radial artery. Unsuccessful recanalization may be caused by incorrect compression or insufficient compression force. Also, too short periods of compression may be causative in this context [24]. According to the work of Tian et al., compression can be performed up to 6 hours with virtually no subjective symptoms [23]. In our work, the clinical manifestations of RAO included the absence of pulsation in a third of the cases and the sensation of paresthesia and numbness of the thumb and index finger. During the ipsilateral compression, the symptoms of ischemia of the hand were not found in any patient. At the same time, Nagai et al. reported that 14% of patients complained of pain in the hand, but this is due to the fact that the researchers used this technique for more than 3 hours [23].

From a pathophysiological perspective, the cause of acute postcatheterization occlusion is occlusive thrombosis [25]. Thrombosis occurs at the site of injury of the vascular wall. In the area of local injury of the vascular wall and slow blood flow during hemostasis, a soft red blood clot easily forms. At this stage, spontaneous restoration of the radial artery patency is possible. Later after retraction and compaction of the red thrombus, the probability of its recanalization goes down. Heparin contributes to the spontaneous recanalization of soft fresh blood clots, its effect just about 4 hours after the procedure [26]. So, rapid restoration of blood flow could be in the first hours after the development of occlusion. Early removal of the compression bandage independently increases the likelihood of restoration of blood flow in the radial artery. In the same line of argument, it will be proper to measure that “prophylactic” ipsilateral ulnar compression may have the highest efficacy in “preventing” RAO, as proven by randomized and nonrandomized data [18, 27].

Ultrasound is the gold standard for the detection of the RAO and the most accurate method, which allows evaluating the flow rate, its direction (antegrade or retrograde), and the arteries' anatomy. Flow absence along with information about anatomy (curves, loops, etc.) is very important for future interventions [27–29]. However, the oximetry/plethysmography test is a simple and inexpensive bedside method. This method is highly sensitive and can be equally useful in assessing of the radial artery patency [6, 7, 16, 18, 29]. We found that rates of RAO by oximetry/plethysmography and duplex ultrasound were equal.

The incidence of bleeding (0.8%) and local hematoma (2.2%) in both groups was comparable. The reduction of the duration of hemostasis did not lead to an increase in bleeding complications and hematomas, compared with the other studies [4, 6, 9, 16, 18, 21].

Radial artery hemostasis is an important issue in the transradial approach since the rate of radial artery occlusion is not negligible in the clinical practice. However, it should be clearly stated that dedicated hemostatic devices, although expensive, associated with patent hemostasis technique are the gold standard in order to prevent RAO [29].

The safety and simplicity of the ipsilateral ulnar artery compression, with the lack of need for complex equipment, combined with its highly significant efficacy and safety decrease the incidence of RAO. This fact could encourage most interventional cardiologists to embrace the technique as default protocol, in addition to other established best practices to reduce RAO after transradial access.

## 5. Limitations

Our study did not include patients with acute coronary syndrome; this does not allow extending the conclusions of this work to this cohort of patients. Our investigation is a single-center study with a limited number of patients. Further research is needed to investigate methods of recanalization of acute occlusion of the radial artery. Early RAO was diagnosed by the oximetry/plethysmography test; an ultrasound examination of these patients was performed only on the following day.

## 6. Conclusion

Shorter hemostasis was associated with significantly less RAO compared to prolonged hemostasis. Rescue radial artery recanalization by one-hour ipsilateral ulnar artery compression was effective only in the short hemostasis group. It remains possible that even more prolonged use (> 1 h) of ipsilateral ulnar artery compression could have a better result on the RAO recanalization rate.

## Figures and Tables

**Table 1:** Baseline characteristics.

	Prolonged hemostasis (*n* = 503)	Short-time hemostasis (*n* = 495)	*p*
Age	61.6 ± 9.9	61.1 ± 9.0	0.43
Height	172.9 ± 8.0	172.9 ± 6.6	0.77
Weight	84.1 ± 11.06	83.9 ± 13.6	0.85
Body mass index	28.1 ± 3.8	28.1 ± 2.9	0.96
Smoking	131 (26%)	122 (24%)	0.69
Diabetes mellitus	106 (21%)	121 (24%)	0.27
Dyslipidemia	134 (27%)	149 (30%)	0.33
Male	367 (73%)	341 (69%)	0.21
Arterial hypertension	448 (89%)	456 (92%)	0.12
Dose of heparin, 1000 IU	7.42 ± 2.92	7.53 ± 2.73	0.42
Diameter of radial artery	2.32 ± 0.38	2.3 ± 0.5	0.58
Body surface area	2 ± 0.19	2 ± 0.16	0.95
PCI	252 (50%)	248 (50%)	1.0

**Table 2:** Periprocedural characteristics.

	Prolonged hemostasis (*n* = 503)	Short-time hemostasis (*n* = 495)	*p*
Crossover	4 (0.8%)	6 (1.2%)	0.73
Fluoroscopy time, min	10.2 ± 10	10.1 ± 9.3	0.98
Procedure time	23.8 ± 10.8	23.7 ± 10.1	0.84
Radial artery spasm	30 (6%)	31 (6.2%)	0.93
Artery/sheath ratio	0.89 ± 0.1	0.89 ± 0.1	0.76

**Table 3:** Frequency of complications.

	Prolonged hemostasis (*n* = 503)	Short-time hemostasis (*n* = 495)	*p*
Rate of RAO after removal	51 (10.1%)	16 (3.2%)	<0.001
Rate of 24-hour RAO	51 (10.1%)	7 (1.4%)	<0.001
Bleeding	4 (0.8%)	4 (0.8%)	1.0
Hematoma (stage 1-2)	11 (2.2%)	11 (2.2%)	1,0
Hematoma (≥ stage 3)	1 (0.2%)	2 (0.4%)	1.0

## Data Availability

The data used to support the findings of this study are available from the corresponding author upon request.

## Abbreviations

RA: Radial access
PCI: Percutaneous coronary interventions
CAG: Coronary angiography
RAO: Radial artery occlusion
UFH: Unfractionated heparin.
